# Effect of adalimumab on anxiety-depression-like behaviors and learning in rats receiving cisplatin

**DOI:** 10.1515/med-2026-1406

**Published:** 2026-06-26

**Authors:** Durmuş Ali Aslanlar, Mehmet Öz, F. Hümeyra Yerlikaya Aydemir, K.Esra Nurullahoglu Atalik

**Affiliations:** Department of Medical Pharmacology, Faculty of Medicine, Necmettin Erbakan University, Konya, Türkiye; Department of Physiology, Faculty of Medicine, Aksaray University, Aksaray, Türkiye; Department of Medical Biochemistry, Faculty of Medicine, Selcuk University, Konya, Türkiye

**Keywords:** cisplatin, adalimumab, anxiety, depression, learning and memory

## Abstract

**Objectives:**

To investigate the neuroprotective effects of adalimumab against cisplatin-induced cognitive impairment (CICI) and to evaluate its potential to ameliorate anxiety- and depression-like behaviors as well as learning and memory deficits through modulation of Tumor necrosis factor-α (TNF-α)–mediated neuroinflammation, cholinergic homeostasis, and apoptosis.

**Methods:**

Adult male Wistar rats were divided into four groups (n=6/group): Control, Cisplatin (2 mg/kg/day, i.p., 10 days), ADA (10 mg/kg, i.p., three doses), and Cisplatin + ADA. Anxiety-, depression-like behaviors and memory performance were assessed using the open field test, elevated plus maze, forced swim test, and novel object recognition test. Serum and hippocampal TNF-α, nitric oxide (NO), acetylcholinesterase (AChE), acetylcholine (ACh), and p53 levels were measured by ELISA.

**Results:**

Cisplatin induced anxiety- and depression-like behaviors and impaired recognition memory without affecting locomotor activity. These behavioral alterations were accompanied by increased TNF-α, NO, AChE, and p53 levels in the hippocampus. ADA treatment significantly reversed behavioral deficits and normalized inflammatory, cholinergic, and stress-related markers.

**Conclusions:**

Adalimumab attenuates cisplatin-induced cognitive and mood disturbances, likely through modulation of TNF-α–mediated neuroinflammation, cholinergic imbalance, and stress-related signaling pathways.

## Introduction

Biomedical research into cancer chemotherapy has significantly improved the survival rates of cancer patients, but a significant proportion of people undergoing chemotherapy suffer from cognitive impairment as a result of the use of chemotherapeutic agents, leading to a significant reduction in their overall quality of life [1]. Chemotherapy-induced cognitive impairment (CICI), also known as ‘chemobrain,’ has been well documented in medical literature. It is a recognized phenomenon that refers to the pathological effects of chemotherapy on cognitive status [2]. A comprehensive understanding of the molecular basis of CICI is crucial for reducing or eliminating cognitive dysfunction during cancer treatment, but the complex mechanism of CICI is not yet fully understood. Undoubtedly, experimental research will play a crucial role in achieving this goal. Cisplatin, a platinum compound, is used as a treatment option for various types of cancer, such as sarcomas, solid tumors in the head and neck region, and ovarian and testicular malignancies, as well as cancers affecting muscles, bones, and blood vessels [3]. Cisplatin administration in rats led to heightened anxiety, compromised motor coordination, and diminished learning and memory capabilities. These effects were linked to neuroinflammation, disturbance of cholinergic balance, and heightened apoptosis [4]. In addition, cisplatin caused an increase in the expression of apoptotic markers and the release of proinflammatory cytokines like TNF-alpha in the mouse hippocampus [5]. Cisplatin administration in male rats resulted in elevated levels of oxidative stress in the hippocampus, induced neuroinflammation, and reduced expression of brain-derived neurotrophic factor (BDNF), leading to cognitive deficits [6]. Altogether, it is understood that the contribution of inflammation, oxidative stress, and increased apoptosis is important in the neurotoxic effect of CICI [4], [5], [6], [7]; therefore, it can be predicted that it may be reasonable to target these factors in the treatment strategies to be developed.

Adalimumab (ADA) is a monoclonal human antibody that belongs to the immunoglobulin G1 class and specifically targets TNF-α. It exerts its effect by inhibiting TNF-α binding to its own receptors or cell surface receptors [8]. The clinical use of Ada in pathologies such as chronic inflammatory rheumatic diseases [9], ulcerative colitis [10], psoriasis, and psoriatic arthritis [11] has been ongoing for many years. Furthermore, the therapeutic and protective efficacy of Ada has been shown in different pathologies such as diabetic nephropathy [12], experimental nerve injury [13], ovarian [14] and renal [15] ischemia reperfusion injury, the experimental acute pancreatitis model [16], methotrexate-induced spleen toxicity [17], the rat osteoarthritis model [18], and the corneal neovascularization model [19]. There are few reports showing the neuroprotective activity of ADA in experimental studies. In the Alzheimer’s mouse model [20] and chronic cerebral hypoperfusion in rats [21], ADA improved cognitive impairments associated with these pathologies. In studies conducted in our laboratory, we have shown that ADA protects against methotrexate-induced mood disorders [22] and sepsis-induced cognitive and mood disorders [23]. Chronic systemic inflammation, characterized by an increase in cytokines like TNF-alpha, plays an important role in the development of dementia. A recent meta-analysis reported that treatment with biological anti-TNFs reduced dementia, with adalimumab showing the second-best effect among these drugs [24]. Given the pivotal role of TNF-α in neuroinflammation and the established anti-inflammatory efficacy of adalimumab, this study was designed to repurpose adalimumab for the management of chemotherapy-induced cognitive impairment. We hypothesized that adalimumab exerts neuroprotective effects by inhibiting TNF-α–mediated pathways and thereby attenuates cisplatin-induced cognitive dysfunction. Accordingly, this study addresses whether adalimumab treatment ameliorates anxiety- and depression-like behaviors as well as learning and memory deficits in a cisplatin-induced CICI model through modulation of neuroinflammation, cholinergic homeostasis, and apoptosis.

## Materials and methods

### Experimental animals

The Necmettin Erbakan University Experimental Medicine and Administration Center provided 24 male Wistar albino rats weighing 330 ± 20 g. The rats were kept in controlled environments with a temperature of 20 ± 2 °C, humidity of 50 ± 10 %, and a 12 h light/dark cycle. Tap water and rat food were provided to the animals without any limitations. The study was conducted at the Necmettin Erbakan University Experimental Medicine and Administration Center in Konya, Turkey. Twenty-four animals were sorted into four groups at random: Control (n=6), Cisplatin (n=6), Adalimumab (ADA; n=6), and Cisplatin + ADA (n=6).

### Experimental protocol

Control (C) group: For 10 days, the rats were given a daily intraperitoneal (i.p.) injection of 0.9 % saline.

Cisplatin (CIS) group: Animals received 2 mg/kg/day, i.p cisplatin for 10 days.

Adalimumab (ADA) group: Animals were given 10 mg/kg ADA, i.p three times during the study: five days before the administration of cisplatin, on day zero and fifth day of cisplatin administration.

Cisplatin plus Adalimumab (CIS + ADA) group: Animals were received 2 mg/kg/day, i.p cisplatin for 10 days and 10 mg/kg, i.p adalimumab, five days before the administration of cisplatin, on day zero and on the fifth day of cisplatin administration.

Cisplatin, obtained from Sigma (St. Louis, MO, USA), was freshly prepared prior to administration, while ADA (Humira©) was sourced from Vetter Pharma-Fertigung GmBH&Co.KG, Ravensburg, Germany. The dosages and administration routes for cisplatin and ADA were selected based on prior research [25], 26]. The animals’ body weights were recorded at both the start and end of the experiments. Behavioral assessments began on the 7th day of cisplatin treatment. Four hours following the last behavioral test on the 10th day, all rats were euthanized with a high dose of anesthesia, and blood and brain Tissue samples were gathered for examination. Behavioral scoring was performed manually by an investigator blinded to the experimental groups. For the FST, immobility was recorded as the cumulative time spent immobile.

### Blood and brain tissue sampling

Four hours following the last behavioral test session, all rats were euthanized via cervical dislocation following blood collection through cardiac puncture under ketamine-xylazine anesthesia. Immediately after, the blood samples were cold-centrifuged for 20 min at 2,000 rpm, and the plasma was separated for oxidative stress marker biochemical analysis. After the brains were removed, the hippocampi were carefully dissected and put right into liquid nitrogen. Following that, the samples were kept at −80 °C for additional analysis.

### Behavioral studies

Tests of behavior were carried out from 9 a.m. to 5 p.m. The rats were given 30 min to adjust to their new surroundings and lessen stress in their home cages in the testing room before any testing began. Between experiments, excrement was removed from all testing equipment and cleaned with 75 % ethanol.

#### Open field test (OFT)

The OFT was employed to assess locomotor activity, exploratory behavior, and anxiety levels. The setup consisted of black-painted plexiglass measuring 80 × 80 cm, with walls 40 cm high. Each rat was gently placed in the center of the open field and allowed to explore freely for 5 min. Recorded measures included the number of rearings, crossings, and the time spent in the center of the arena. The apparatus was thoroughly cleaned between tests to remove any odor-related influences on behavior.

#### Elevated plus maze test (EPMT)

The EPMT was used to measure anxiolytic activity, and it was done right after the OFT. The EPMT comprised a core square (10 × 10 cm), two open arms (50 × 10 cm), two closed arms (50 × 10 cm), and a cross-shaped maze with an open top. The labyrinth was raised to a height of 50 cm. For 5 min, each rat was left to freely explore the maze after being placed on the central square with its arms open. An entry into an arm was recorded when all four paws of the rat crossed into the arm. The number of entries into the open arms and the total time spent in these arms were measured and analyzed statistically.

#### Forced swimming test (FST)

The FST was utilized to evaluate “behavioral despair,” a model believed to mirror human depression. The procedure was based on the protocol by Caletti et al. (2012) [27], with slight modifications. It included a 15 min training session, followed by a 5 min test session 24 h later. During the training, rats were placed in cylindrical plexiglass tanks (25 × 60 cm) filled with 35 cm of water at a temperature of 25 ± 1 °C, allowing them to swim freely. In the test session, the rats’ behaviors were videotaped for later ethological analysis. An experienced observer, blind to the experimental groups, evaluated the immobility behavior. Immobility was defined as the absence of movement needed to keep the head above water. The tanks were emptied and thoroughly cleaned between each animal.

#### Novel object recognition test (NORT)

The NORT was performed in a Plexiglas^®^ open field chamber measuring 60 × 50 × 25 cm^3^. The experiment took place over three days. On the first day, each rat was placed in the empty open field box for 5 min to get accustomed to the environment before any objects were introduced. On the second day, two distinct objects of equal significance were positioned 48 cm apart in opposite corners of the box, and the rats were allowed to explore for 5 min. One of the previously used objects (the familiar object) and a new object were placed at opposite corners of the box, 48 cm apart, an hour later. The rodents spent a further 5 min investigating the box. The following formula was used to calculate the discrimination index: (Time with novel object – Time with familiar object)/(Total exploration time).

### Biochemical analysis

Serum levels of TNF-α (catalog number: 201-11-0765), as well as concentrations of TNF-α (catalog number: 201-11-0765), P53 (catalog number: 201-11-1643), NO (catalog number: 201-11-0704), AChE (catalog number: 201-11-0725), and ACh (catalog number: 201-11-0723) in the brain, were assessed using a commercially available enzyme-linked immunosorbent assay (ELISA) kit from Shanghai Sunred Biological Technology Co. (China). This kit employs a two-site sandwich assay method, utilizing two distinct polyclonal antibodies that bind to different epitopes of the targeted proteins, including rat TNF-α, brain TNF-α, P53, NO, AChE, and ACh.

### Statistical analysis

Statistical analyses were conducted using SPSS for Windows (version 22.0) and GraphPad Prism (version 9.0). Data are presented as mean ± standard deviation, and error bars represent the standard deviation. Prior to comparative analyses, the assumptions of normality and homogeneity of variances were assessed. One-way analysis of variance (ANOVA) was used to evaluate differences among the treatment groups. When a significant overall effect was observed, post hoc multiple comparisons were conducted using the Tukey honestly significant difference (HSD) test. A p-value of less than 0.05 was considered statistically significant.

A total of 24 rats were included in the study, with 6 animals allocated to each experimental group. The sample size was determined based on previous studies with similar experimental designs and ethical considerations to minimize animal use, using an *a priori* power analysis conducted with G*Power software (version 3.1). For the power analysis performed for the one-way analysis of variance, the significance level was set at α=0.05 and the statistical power was set at 80 % (power=0.80).

**Ethical statement:** This study was approved by the Necmettin Erbakan University Animal Experiments Local Ethics Committee (approval number: 2021-043) and adhered to the guidelines set forth by the National Institute of Health Guide for Care and Use of Laboratory Animals (Publication No. 85-23, revised 1985).

## Results

Body mass measured at the beginning of the research was found to be 330.6, 332.2, 340.9 and 336.9 g in the control, CIS, ADA, and ADA + CIS groups, respectively (p>0.05). At the end of the research, although a decrease in body mass was observed in the cisplatin administered group, there was no statistical significance among the groups (p>0.05, data not presented).

### Locomotion-related behavior

**Figure 1:**
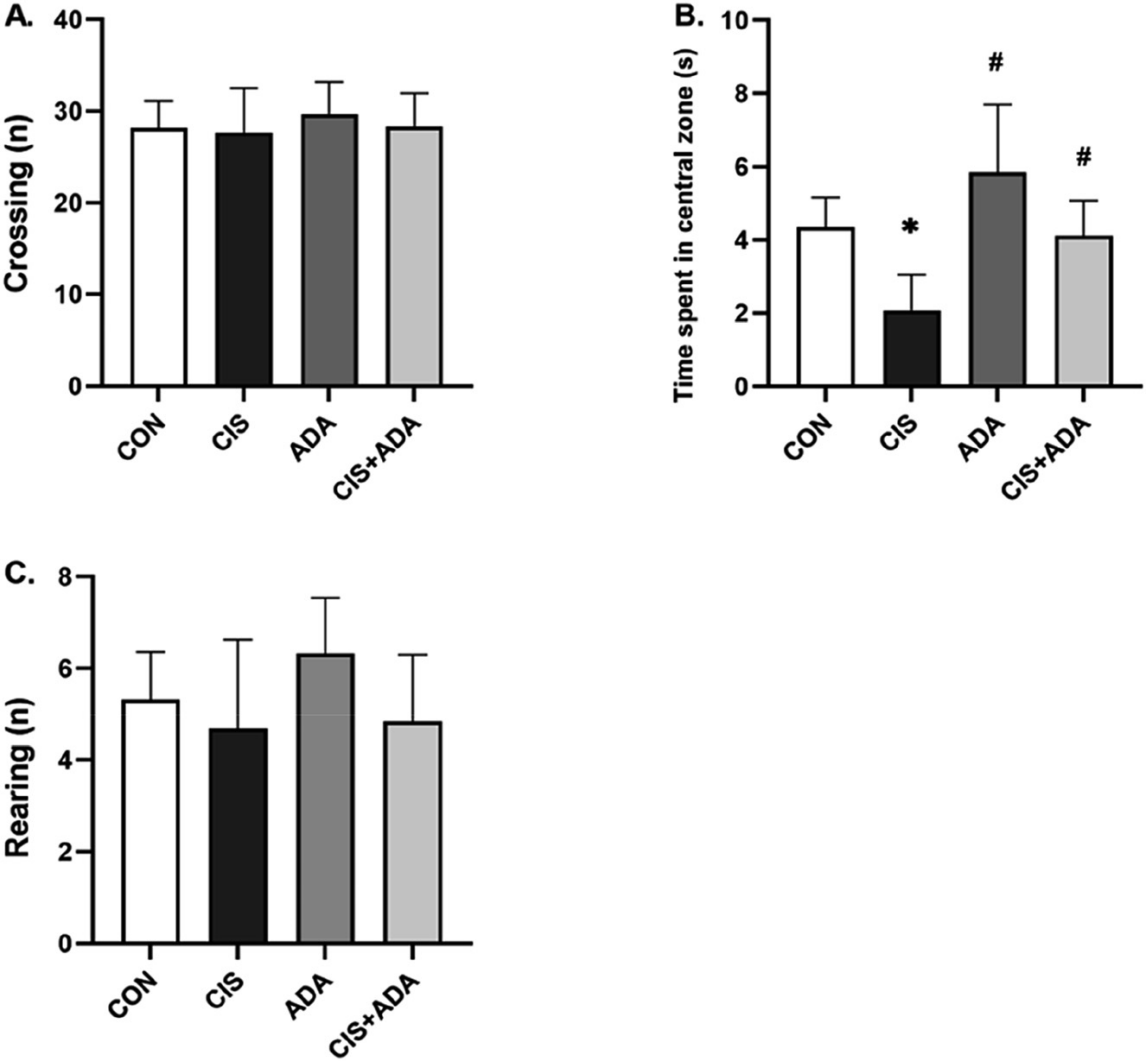
Open field test. (A) Crossing count, (B) time spent in central zone (second), (C) rearing count. CON, Control; CIS, Cisplatin; ADA, Adalimumab; CIS + ADA, Cisplatin + Adalimumab. Data were expressed as mean ± SD (n=6). ^*^p<0.05 vs. the control and ^**#**^p<0.05 vs. the cisplatin group.

The crossing number in the OFT was used for locomotor activity. As indicated in Figure 1A, the locomotor activity did not vary significantly among the experimental groups (p>0.05). This indicates that rats in all groups in the study had similar locomotor activity. Therefore, it is accepted that the changes they show in the behavioral tests are not due to the difference in motor skills but to the impact of the drugs utilized in the study.

### Anxiety-like behaviors

Anxiety-like behaviors were evaluated using two tests: the OFT and the EPMT. In the OFT, the amount of time spent in the central zone was used as an anxiety measure. Rats treated with cisplatin spent substantially less time in the central zone than the control group (Figure 1B, p<0.05). Treatment with ADA improved the time spent in the central zone, which had been reduced by cisplatin (p<0.05). While ADA alone extended the time spent in the central zone (p<0.05), no significant difference was observed in comparison to the control group (p>0.05). There were no significant differences in the number of rearings, another anxiety indicator, found between the groups in the OFT (Figure 1C, p>0.05).

**Figure 2:**
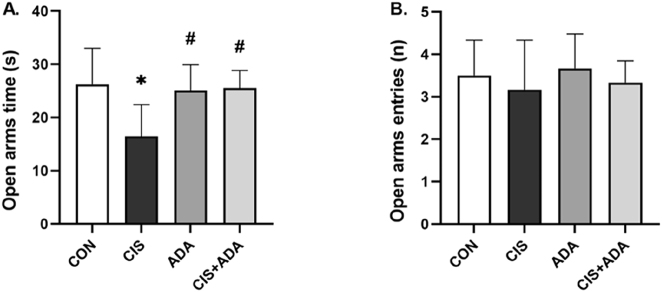
Elevated plus maze test. (A) Open arms time (second), (B) open arms entries count. CON, Control; CIS, Cisplatin; ADA, Adalimumab; CIS + ADA, Cisplatin + Adalimumab. Data were expressed as mean ± SD (n=6). *p<0.05 vs. the control and ^**#**^p<0.05 vs. the cisplatin group.

In the EPMT, which also assesses anxiety-like behaviors, comparisons were made between the groups based on the number of entries into the open arms and the total time spent there (Figure 2A and B). There were no notable differences in the number of entries into the open arms among the groups (p>0.05). However, the total time spent in the open arms was considerably lower in the cisplatin group in comparison to all other groups (Figure 2A, p<0.05), and ADA treatment effectively normalized this effect induced by cisplatin.

### Depression-like behaviors

**Figure 3:**
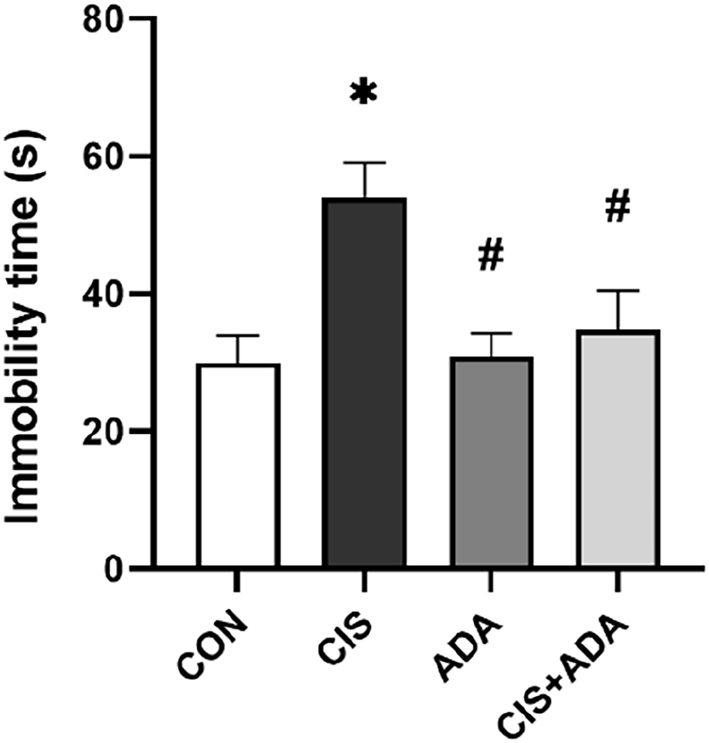
Forced swim test. Immobility time (second). CON, Control; CIS, Cisplatin; ADA, Adalimumab; CIS + ADA, Cisplatin + Adalimumab. Data were expressed as mean ± SD (n=6). *p<0.05 vs. the control and ^**#**^p<0.05 vs. the cisplatin group.

Depression-like behaviors were assessed with the FST, which included training and testing phases. During the training phase, all rats swam freely in the water tank for 15 min, during which video recording was not taken. After 24 h, rats were permitted to swim for 5 min during the test phase, and video recordings were made. Rats receiving cisplatin showed a longer immobility time in the FST in comparison to all other groups (Figure 3, p<0.05). The immobility time in the control and cisplatin groups was 29.97 ± 1.61 and 54.04 ± 2.05, in that order, and this was statistically significant (p<0.05). Adalimumab treatment reversed this depression-like behavior induced by cisplatin significantly (p<0.05). The immobility time in the ADA-treated group was 34.73 ± 2.35. The rats that received only ADA displayed a similar response with the control.

### Learning and memory assessment

**Figure 4:**
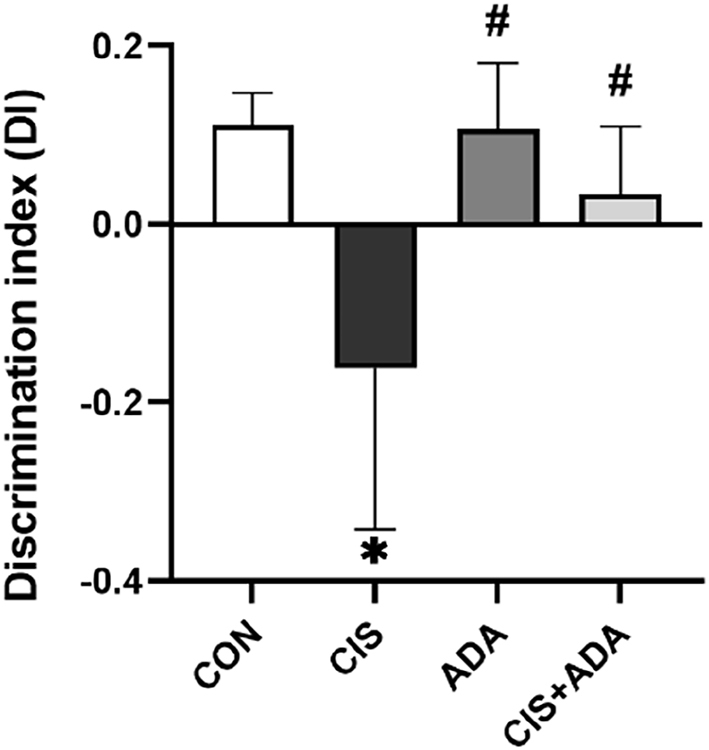
Discrimination index (DI). CON, Control; CIS, Cisplatin; ADA, Adalimumab; CIS + ADA, Cisplatin + Adalimumab. Data were expressed as mean ± SD (n=6). *p<0.05 vs. the control and ^**#**^p<0.05 vs. the cisplatin group.

During the practice phase of the new object recognition test, all rats were permitted to move freely in the open field arena for 5 min on the first day and were not videotaped. After 24 h, during the first part of the testing phase, all rats were found to engage with two identical objects placed at equal lengths at the corners of the open field arena for similar periods of time. In the test phase, 1 h after this phase, one of the identical objects was substituted with a different new object. The affinity of rats treated with cisplatin to the new object did not differ from the familiar object. The recognition index, calculated by assessing time spent with the two objects, was different in cisplatin-treated rats in comparison to all other groups (Figure 4, p<0.05). However, in both the control and ADA-treated groups (both with and without cisplatin), rats were more interested in the new object, and the recognition index was higher than in rats receiving cisplatin (p<0.05).

### Serum and brain TNF-α levels

**Figure 5:**
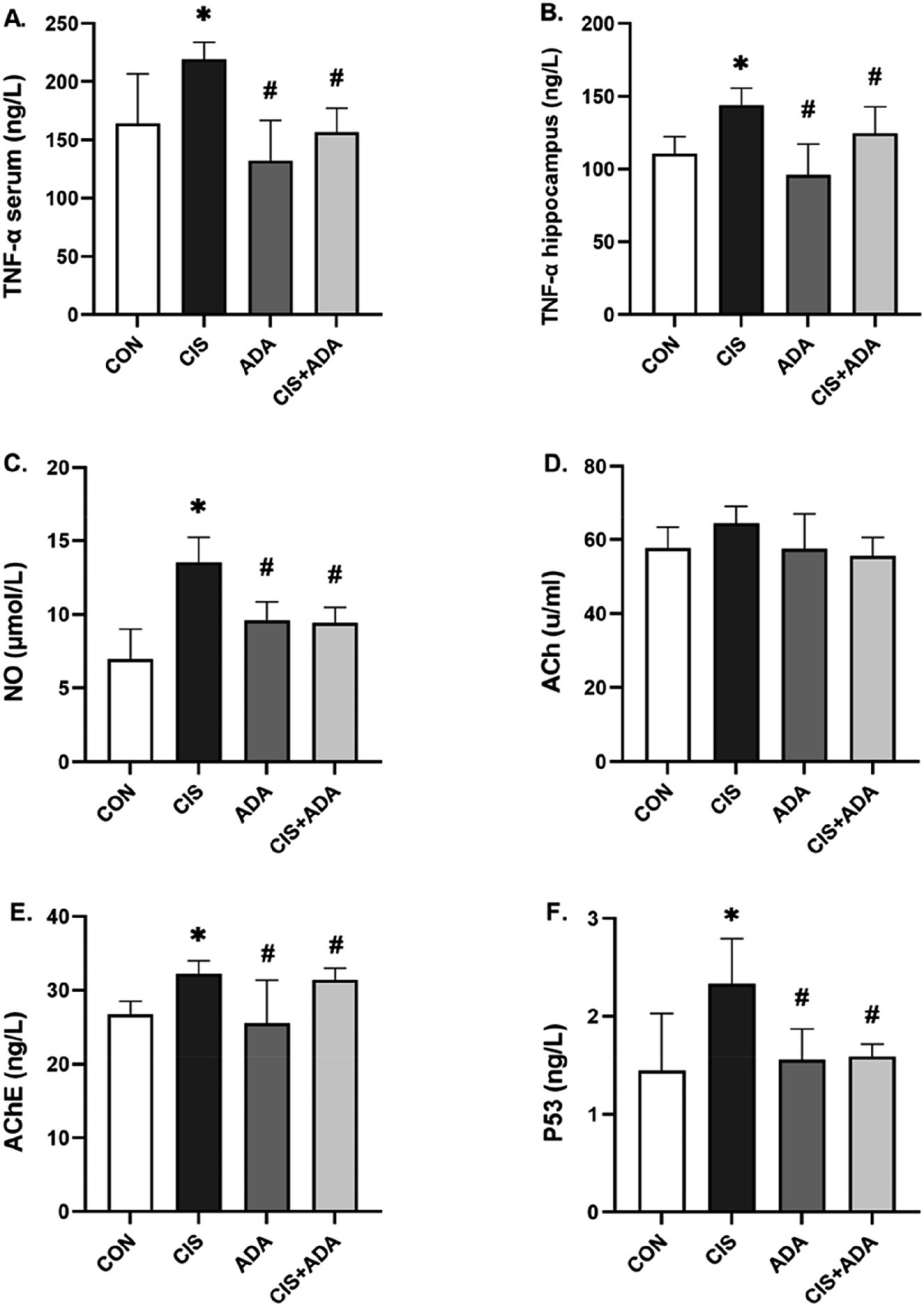
ELISA result for serum TNF-α level. ELISA results for hippocampal TNF-α, NO, ACh, AChE, and p53 levels. CON, Control; CIS, Cisplatin; ADA, Adalimumab; CIS + ADA, Cisplatin + Adalimumab. Data were expressed as mean ± SD (n=6). ^*^p<0.05 vs. the control and ^**#**^p<0.05 vs. the cisplatin group.

In our study, we evaluated the effect of cisplatin on the inflammatory process both systemically (in the blood serum) and locally (in the brain hippocampal tissue) using TNF-α, an important proinflammatory cytokine. Cisplatin caused inflammation in both blood and hippocampal tissue. It resulted in increased TNF-α levels in serum (Figure 5A) and hippocampus (Figure 5B) tissue compared with the control group (p<0.05). Treatment with ADA, a TNF-α inhibitor, ameliorated this cisplatin-induced increase in TNF-α levels (p<0.05). In rats receiving only ADA, TNF-α levels were reduced in both serum and brain tissue in comparison to the control group; however, this reduction was not statistically significant (p>0.05).

### Brain nitric oxide (NO) level

Treatment with cisplatin led to an increase in NO levels in the hippocampus of rats (Figure 5C, p<0.05). One of the key findings of our research indicates that ADA ameliorated the increased hippocampal NO levels caused by cisplatin (Figure 5C, p<0.05). There was no significant difference in the levels of NO in the tissue of the hippocampus between the rats that were treated with ADA alone and the control group (p>0.05).

### Brain ACh and AChE activity

Although cisplatin caused a slight increase in hippocampal tissue ACh levels (Figure 5D, p>0.05), these changes did not reach significance. Similarly, no difference was found in the changes induced by ADA alone (p>0.05). However, cisplatin treatment caused an increase in brain hippocampal tissue AChE activity compared to control rats (Figure 5E, p<0.05). The effect of ADA was to ameliorate the cisplatin-induced increase slightly (Figure 5E, p>0.05).

### Brain p53 protein levels

Brain p53 protein levels exposure to cisplatin markedly elevated p53 protein levels compared to the control group, suggesting an upregulation of stress-responsive pathways associated with potential cellular damage. However, co-administration of ADA counteracted these effects in cisplatin-treated rats, significantly reducing the expression of this pro-apoptotic marker (Figure 5F, p<0.05).

## Discussion

Cognitive impairment caused by chemotherapy drugs has been extensively documented and reported in both human and experimental research, and this study, in agreement with the literature, reconfirmed that cisplatin causes mental and cognitive impairment in rodents. In our protocol, cisplatin caused neuroinflammation, disturbed cholinergic homeostasis, and triggered apoptosis. However, the neuroprotective effect of ADA, a TNF-alpha inhibitor, in this cisplatin-induced pathology was demonstrated for the first time. As expected, ADA effectively prevented both systemic inflammation and neuroinflammation. Furthermore, regulation of cholinergic impairment and prevention of apoptosis are important factors contributing to ADA’s neuroprotective effect and constitute the most important findings of our study.

Our results revealed that cisplatin treatment caused cognitive and mood disturbances in male rats but did not lead to any substantial alterations in locomotor activity (Figure 1A). Cisplatin treatment decreased the time spent in the central zone during the OFT and in the open arms during the EPM test, both of which are indicative of increased anxiety (Figures 1B and 2A). It also caused an increase in immobility time in the forced swim test (Figure 3), as well as a negative discrimination index in the NOR test (Figure 4). Neuroinflammation is crucial in the mechanism of effect of cisplatin chemotherapy, leading to cognitive and mood disorders, and numerous reports have shown that elevated TNF-alpha is associated with these inevitable complications [4], 25], 26]. Consequently, we postulated that TNF-alpha inhibition might mitigate the neurotoxicity induced by cisplatin.

To test this hypothesis, we employed ADA, a biological inhibitor of TNF-alpha. Although ADA possesses limited blood-brain barrier (BBB) permeability due to its physical characteristics [28], it mitigated not only systemic inflammation but also neuroinflammation, as evaluated in hippocampal tissue. This neuroprotective efficacy, despite the restricted CNS penetration, can be elucidated by the systemic regulation mechanism: While the ability of cisplatin to traverse the BBB remains a subject of debate [29], it is well documented that it induces the systemic production of pro-inflammatory cytokines such as TNF-alpha [30]. These systemically derived cytokines can compromise BBB integrity via circulation and propagate neuroinflammation [31]. Thus, the reduction of systemic inflammation may restrict the passage of cytokines across the BBB, thereby indirectly averting cognitive decline [23]. In the present study, cisplatin administration provoked both systemic and neuroinflammation (Figure 5A and B). While the reduction in serum TNF-alpha following adalimumab treatment was anticipated, the concomitant decrease in hippocampal TNF-alpha levels serves as a compelling indication that systemically produced TNF-alpha is a critical driver of cisplatin-induced neuroinflammation. Consistent with our observations in LPS-induced cognitive impairment [23], we have reinforced the concept that systemic inflammation acts as a precursor to neuroinflammation and subsequent cognitive decline. This mechanism suggests a potential therapeutic strategy for managing such complex disorders.

The cholinergic system has a significant impact on mechanisms of learning and memory. The degeneration of cholinergic neurons in the basal forebrain and hippocampus causes a deterioration in cholinergic functioning, which in turn causes memory impairment, according to the cholinergic theory of AD [32]. In chemotherapy-induced neurotoxicity, increased AChE activity is associated with cognitive decline, indicating increased breakdown of acetylcholine, a neurotransmitter that serves a crucial role in learning and memory formation. The cisplatin treatment protocol applied in our study caused an increase in hippocampal tissue AChE activity in rats, in accordance with the literature (Figure 5E) [4], [33], [34], [35]. Cisplatin also stimulates the release of inducible nitric oxide synthase in the hippocampus, which is linked to a rise in AChE activity [34]. The most interesting finding of our research indicates the administration of ADA attenuated the cisplatin-induced increase in hippocampal AChE activity in rats (Figure 5E). As far as we know, we were the first to show the impact of ADA on hippocampal AChE activity in a mouse model of Alzheimer’s disease induced by LPS [23], and here we report the same effect in rats. One of the complications that has an important place in the pathophysiology of cisplatin-induced cognitive impairment is the changes in the apoptotic pathway, which is not surprising for an anti-cancer drug. Cisplatin treatment causes a rise in the levels of pro-apoptotic markers like caspase 3, bax, and p53 in rodents, while inhibiting anti-apoptotic markers such as bcl-2 [4], 25], 35], 36]. In our study, cisplatin administration led to a significant increase in the p53 level in the hippocampal tissue of rats compared to the control group (Figure 5F). Adalimumab treatment significantly attenuated the cisplatin-induced elevation of p53, which is a key upstream regulator of cellular stress and apoptosis. Given that p53 activation is a pivotal trigger for downstream apoptotic cascades, this reduction suggests that adalimumab modulates the early pro-apoptotic signaling initiated by cisplatin. Although our study is the initial report demonstrating the impact of ADA on p53 levels in hippocampal tissue, apoptotic effects of ADA have been demonstrated in pancreatic tissue in the acute pancreatitis model [16], ovarian tissue in the ischemia-reperfusion injury model [14], and microglia in the LPS-mediated inflammation model [37]. In contrast, another study reported that ADA was found to decrease the production of proinflammatory cytokines via inducing apoptosis in human monocytic cell line cells [38]. All together, this study showed promising effects of ADA on cognitive impairment in a CICI model, but our study has some limitations. First, oxidative stress markers, histopathological changes (including glial activation), and the protective effects of lower ADA doses were not evaluated. Second, the exclusive use of male rats limits the generalizability of our findings regarding sex-dependent mechanisms. Regarding the apoptotic pathway, it should be noted that while p53 provides evidence of pro-apoptotic stress, the lack of data on downstream executioner markers (e.g., cleaved caspase-3 or TUNEL staining) limits a definitive conclusion regarding the full reversal of the apoptotic process. Finally, although we propose a peripheral regulation mechanism, the precise pathways underlying ADA’s neuroprotection despite its limited BBB permeability require further elucidation.

## Conclusions

In this research, we illustrated the ameliorative effect of ADA, a TNF-alpha inhibitor, on anxiety- and depression-like behaviors, as well as learning-memory activity in a cisplatin-induced CICI model. Cisplatin caused neuroinflammation and disrupted cholinergic and balance while upregulating p53-mediated stress markers. ADA significantly improved cognitive and mood disorders by modulating these alterations. Additional research should focus on elucidating the mechanism behind ADA’s effect on regulatory and apoptotic-related signaling within the cholinergic system in brain tissue.
